# The Impact of Different Cooking Methods on the Flavor Profile of Fermented Chinese Spicy Cabbage

**DOI:** 10.3390/molecules28186539

**Published:** 2023-09-09

**Authors:** Huamin Li, Hui Guan, Xiru Zhang, Shaohua Xing, Wenli Liu, In-Cheol Kim, Hansheng Gong

**Affiliations:** 1 School of Food Engineering, Ludong University, Yantai 264025, China; hmli@ldu.edu.cn (H.L.);; 2 Yantai Key Laboratory of Nanoscience and Technology for Prepared Food, Ludong University, Yantai 264025, China; 3 Yantai Engineering Research Center of Green Food Processing and Quality Control, Ludong University, Yantai 264025, China; 4 Department of Food Engineering, Mokpo National University, Jeonnam 534729, Republic of Korea

**Keywords:** boiling, stir-frying, flavor, sensory, Chinese spicy cabbage

## Abstract

Chinese spicy cabbage (CSC) is a common traditional fermented vegetable mainly made of Chinese cabbage. In addition to eating raw, boiling and stir-frying are the most common cooking methods for CSC. To identify the impacts of boiling or stir-frying on the quality of CSC, the physicochemical properties, flavor compounds, and sensory properties of CSC were analyzed. A total of 47 volatile flavor compounds (VFCs) were detected by gas chromatography–mass spectrometry. Sulfide was determined as the main flavor compound of CSC, mainly contributed by cabbage, garlic, and onion odors. The content of sulfide decreased significantly after cooking. Nonanal, geranyl acetate, and linalool were newly generated after boiling with odor activity value (OAV) > 1, and contributed fatty, sweet, fruity, and floral odors to BL-CSC. 1-Octen-3-one, 1-octen-3-ol, octanal, nonanal, and (*E*)-2-nonenal were newly generated after stir-frying with OAV > 1, and contributed mushroom, fatty, and green odors to SF-CSC. Diallyl trisulfide, nonanal, (*E*)-β-ionone, β-sesquiphellandrene, and (*E*)-2-decenal were considered as the potential key aroma compounds (KACs) to distinguish the CSCs after different heat treatment. After cooking, the total titratable acidity of CSC increased and the sensory properties changed significantly. This study provides valuable information and guidance on the sensory and flavor changes of thermal processing fermented vegetables.

## 1. Introduction

Chinese spicy cabbage (CSC, also known as “Chinese cabbage kimchi”) is a traditional fermented vegetable in northeast China and Korea. CSC is usually made from cabbage, scallion, ginger, garlic, red pepper, salt, and other ingredients, and fermented by various microorganisms present in the raw materials [1]. Among these microorganisms, lactic acid bacteria (LAB) played a dominant role in the fermentation process, producing organic acids, amino acids, sugars, and other metabolites including volatile flavor compounds (VFCs), thereby improving the nutritional and sensory properties of vegetables [2]. The CSC is usually eaten directly and served as a side dish or appetizer. Due to continuous fermentation, microbial metabolism and enzyme activity continued after optimal ripening, leading to over-ripening or deterioration mainly manifested as peculiar smell, over-acidity, increased bitterness, softening texture, and packaging expansion during marketing and storage [2,3]. In particular, CSC packaged in sealed pouches produces carbon dioxide and pressure, resulting in volume expansion, package leaking, and breakage [4,5].

Boiling or stir-frying are the most common processing methods for daily consumption of CSC besides direct eating. After heat treatment, the number of microorganisms in CSC decrease significantly, delaying the changes in pH, acidity, organic acid, microbial population, and carbon dioxide, which could stabilize the quality of CSC and effectively maintain its shelf life [5,6]. Therefore, CSC boiled or stir-fried is also the product most frequently sold packaged in sealed pouches on the market. Boiling and stir-frying are two common processing methods in Chinese cuisine, which could affect the nutrition and sensory characteristics, aroma components, and levels of bioactive compounds of vegetables by changing the chemical composition [7,8]. A previous study showed that the content of flavonols and hydroxycinnamic acid decreased in boiled kale [9]. Most phenolic compounds (gallic acid, 3,4-dihydroxybenzoic acid, 1,2-dihydroxybenzene, syringic, caffeic, rutin trihydrate, p-coumaric acid, apigenin-7-glucoside, quercetin, trans-cinnamic acid, and naringenin) in celery root were reduced after boiling [10]. Reis, et al. [11] showed that the content of glutamic acid, arginine, proline, alanine, valine, serine, and glycine in mushroom were significantly reduced after boiling. Boiling resulted in significant loss of ascorbic acid, soluble proteins, and soluble sugars in broccoli [12]. After boiling, cauliflower had an astringent taste, and watermelon juice had a cooking and sulfur odor [13,14]. Stir-frying could also change the nutrition and flavor of foods. The contents of ascorbic acid and chlorophyll in broccoli decreased after stir-frying with soybean oil for 5 min [12]. Dimethyl sulfide, allyl alcohol, diallyl sulfide, methyl allyl disulfide, and diallyl disulfide were identified as the major volatile compounds in stir-fried garlic. Increasing the frying temperature and time lowered the contents of these sulfur compounds [15]. After frying, the content of volatile compounds in adzuki beans increased, and the odorants changed from “green” and “grassy” to “roasted” and “nutty” [16]. Alcohols, aldehydes, ketones, acids, and esters were the main sources of characteristic flavors of stir-frying mutton “sao zi”, generated by lipid hydrolysis and oxidation [17]. Different cooking methods could produce different flavors, which directly affect consumer choice and food industrialization [18]. However, there have been few studies on the flavor profile changes in fermented vegetables after heat processing, and the difference in flavor between boiled and stir-fried CSC was unclear.

In this study, gas chromatography–mass spectrometry (GC-MS), principal component analysis (PCA), odor activity value (OAV), and fold change (FC > 2 or <0.5) were used to investigate the key aroma compounds (KACs) causing flavor differences in CSC after boiling or stir-frying. And the possible generation pathways of KACs were analyzed. In addition, the effects of boiling or stir-frying on the physicochemical and sensory properties of CSC were evaluated. This study provides valuable insights into the impact of cooking on the flavor of CSC.

## 2. Results and Discussion

### 2.1. Physicochemical Properties

After cooking, the total titratable acidity (TTA) concentration increased significantly from 1.57 ± 0.01 g/100 g to 1.63 ± 0.01 g/100 g, which might be due to the increase in the organic acids escaping from the cells after heat treatment [19]. There was no significant difference in total sugar content between boiled CSC (BL-CSC, 2.35 ± 0.25 g/100 g) and the CSC without thermal processing group (CK-CSC, 2.60 ± 0.19 g/100 g), while the total sugar content of stir-fried CSC (SF-CSC) was decreased to 1.72 ± 0.24 g/100 g. The decrease in sugar content could be attributed to the Maillard reaction between reducing sugars and the primary amino group at high temperatures. The stir-frying temperature (180 °C) was significantly higher than the boiling temperature and temperature is one of the main factors affecting Maillard reaction [19].

### 2.2. Variation in VFC Profile

**Figure 1:**
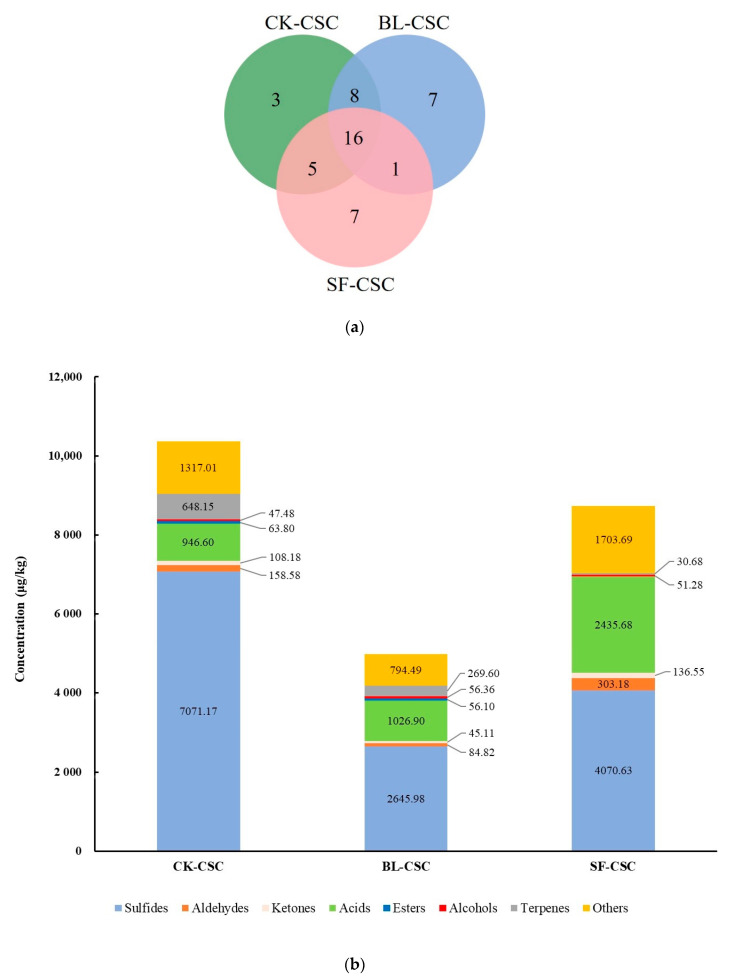
Variation in VFC profile of CSC after cooking. (**a**) VFC Venn diagram. (**b**) VFC concentration of different CSC samples. Abbreviations: VFCs, volatile flavor compounds; CSC, Chinese spicy cabbage; CK-CSC, the CSC without thermal processing group; BL-CSC, boiled CSC; SF-CSC, stir-fried CSC.

The composition and concentration of VFCs in fermented vegetables had an important influence on sensory characteristics and consumer preference [20]. As shown in Table 1, a total of 47 VFCs were detected by GC-MS in CK-CSC, BL-CSC, and SF-CSC, including 15 sulfides, 10 aldehydes, 4 ketones, 3 acids, 4 esters, 4 alcohols, 5 terpenes, 1 nitrile, and 1 phenol. The composition of VFCs was significantly different among the three groups as shown in Figure 1a. In both CK-CSC and BL-CSC groups 32 VFCs were detected, of which 24 compounds were the same. In SF-CSC 29 VFCs were detected, of which 21 compounds were the same as in CK-CSC and significantly different from BL-CSC, and only 17 compounds were the same. In addition to the composition, the concentration of VFCs among the three groups was also significantly different (Figure 1b). The total concentrations of VFCs in CK-CSC, BL-CSC, and SF-CSC were 10,360.97 μg/kg, 4979.36 μg/kg, and 8731.69 μg/kg, respectively. Compared with CK-CSC, the content of sulfides, aldehydes, ketones, and terpenes in BL-CSC decreased dramatically by 62.58%, 46.51%, 58.30%, and 58.40%, respectively. Conversely, alcohols and acids showed increasing trends, with eventual increases of 18.70% and 8.48%, respectively. Compared with CK-CSC, the content of sulfides and terpenes in SF-CSC were reduced by 42.43% and 95.27%, respectively. The content of aldehydes and acids increased obviously by 91.18% and 157.31%, respectively.

**Figure 2:**
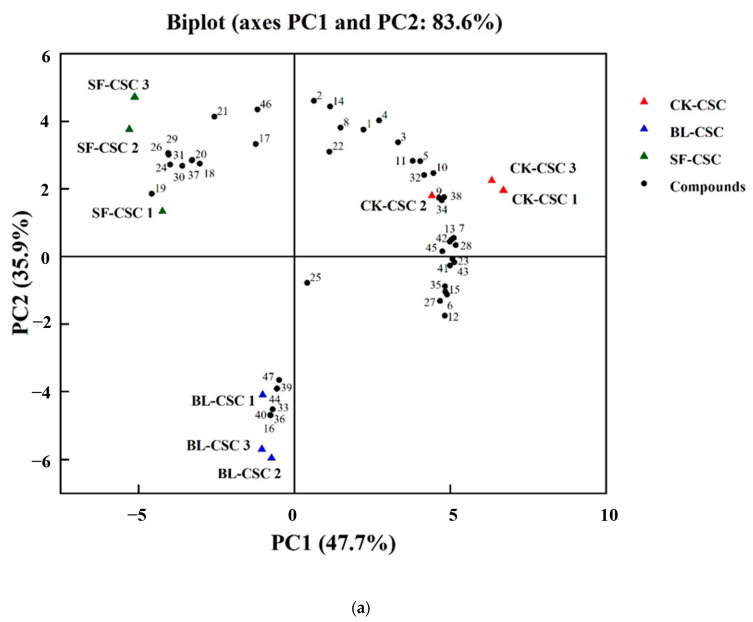

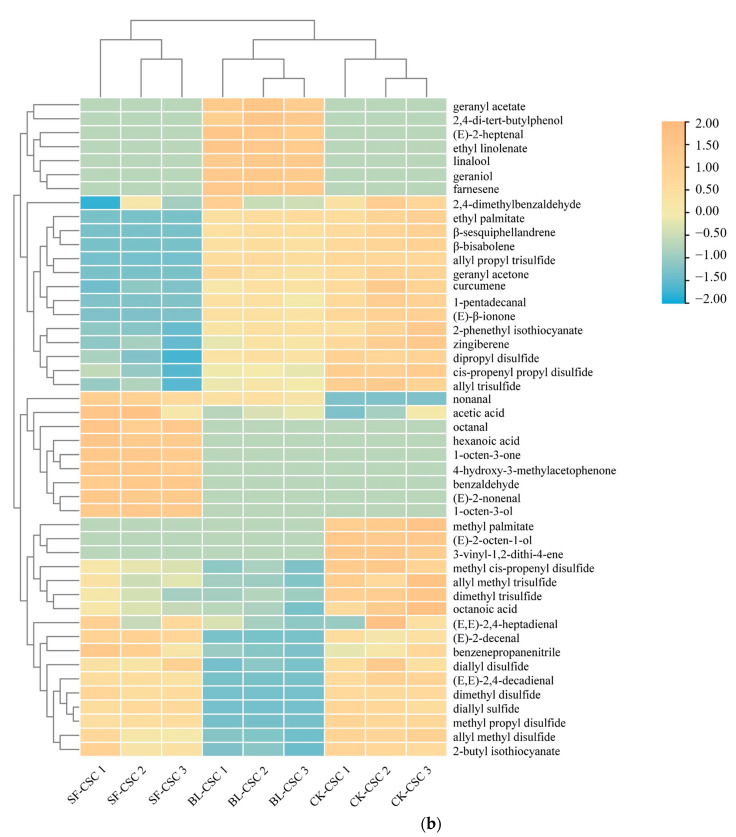
Evolution of flavor compounds in CSC samples under different cooking methods. (**a**) Biplot of the flavor compounds identified from CSC. The number was in accordance with that in Table 1. (**b**) Heatmap analysis of flavor compounds of CSC samples. The color scale represents the concentration of flavor compounds, red indicating high concentration and blue indicating low concentration. Abbreviations: CSC, Chinese spicy cabbage; CK-CSC, the CSC without thermal processing group; BL-CSC, boiled CSC; SF-CSC, stir-fried CSC.

To further investigate the discriminatory VFCs contributing to the CSC after cooking, the PCA was applied. As shown in Figure 2a, PC 1 and PC 2 accounted for 83.6% of the total variation. The VFCs of CK-CSC, SF-CSC, and BL-CSC were diverse and distinct, and they were mainly located in the first, second, and third quadrant, respectively. The PCA confirmed that different cooking methods affected the composition of flavor compounds of CSC. A heatmap was applied to exhibit how the different cooking methods varied in flavor compound composition by applying Euclidean distance as a similarity measure (Figure 2b). The clustering in the dendrogram was similar to the PCA plots (Figure 2a) and the groupings were consistent with the PCA results. The VFCs of CSC after cooking were distinguished.

### 2.3. Screening of the KACs

The odor profiles of CSC not only depend on the composition and concentration of VFCs but are also affected by odor threshold (OT), which determines the OAV value of flavor compounds. In general, a compound with OAV greater than 1 was considered to be the main contributor of aroma, and the contribution and importance of the compound to the aroma increased with the increase in OAV [38]. A total of 21 VFCs with OAV > 1 and definite odor description were selected as the KACs in different CSC samples as shown in Table 1, including 8 sulfides, 6 aldehydes, 2 ketones, 1 acid, 1 ester, 2 alcohols, and 1 terpene.

#### 2.3.1. Sulfides

Sulfides were the most abundant VFCs in the CSC. In total, 15, 11, and 13 sulfides were detected in CK-CSC, BL-CSC, and SF-CSC, respectively. Sulfides were considered to be the most significant volatiles to determine the overall aroma of CSC because of the low threshold [39]. They originated from Chinese cabbage, radish, red pepper, garlic, onion, and ginger [20,40]. The degradation of sulfur-containing amino acids in vegetables was the main source of sulfur-containing compounds [41]. Allicin in garlic was another important source of sulfide compounds in CSC [24]. Sulfides contributed pungent, garlic, onion, and sulfurous aroma to fermented vegetables. As shown in Table 1, eight sulfides with OAV > 1 were determined as the KACs, among which dimethyl trisulfide (OAV = 130,965.66) and allyl methyl disulfide (OAV = 5146.23) were identified as the most important VFCs with the highest OAV value, mainly presenting cabbage and garlic odor, which are the main flavors of CSC.

Compared with CK-CSC, the sulfide content in BL-CSC and SF-CSC significantly decreased by 62.58% and 42.43%, respectively (Figure 1b). Most sulfides were decreased after cooking, and four sulfides including diallyl sulfide, dimethyl disulfide, methyl propyl disulfide, and 3-vinyl-1,2-dithi-4-ene were not detected in BL-CSC, and 3-vinyl-1,2-dithi-4-ene and allyl propyl trisulfide were not detected in SF-CSC (Table 1). Previous studies indicated that sulfides were generated from garlic through thermal degradation of non-volatile compounds and thermal interactions of sugars, lipids, and non-volatile compounds. Sulfide content in cooked or fried crushed garlic was related to the cooking time and frying temperature. The increased frying temperature led to the decrease in sulfide content [15]. A previous study indicated that the di- and tri-sulfides would break down to corresponding thiols and some were lost completely if heated with a proteinaceous food. This might be the main reason for sulfides decreasing after cooking because CSC contains high-protein and amino acid ingredients such as shrimp sauce and fish sauce [42].

#### 2.3.2. Aldehydes

Aldehydes are important characteristic flavor compounds in vegetables, which are generally associated with amino acid catabolism, ketoacid decarboxylation, and unsaturated fatty acid oxidation [43]. As shown in Table 1, a total of 10 aldehydes were identified in three CSC groups. And five, four, and eight aldehydes were detected in the CK-CSC, BL-CSC, and SF-CSC groups, respectively. (*E*,*E*)-2,4-Heptadienal, (*E*)-2-decenal, and (*E*,*E*)-2,4-decadienal were identified as the KACs of CK-CSC with OAVs > 1, which contributed significantly to the nutty, waxy, and fatty flavor. (*E*,*E*)-2,4-Heptadienal (fatty, nutty, green) and nonanal (fatty, citrus, green) contributed greatly to the aroma of BL-CSC. Nonanal and (*E*)-2-heptenal (fatty, almond, green) were newly generated in the BL-CSC. (*E*,*E*)-2,4-Heptadienal, octanal (fatty, fruity, lemon), nonanal, (*E*)-2-nonenal (green, cucumber), (*E*)-2-decenal, and (*E*,*E*)-2,4-decadienal were the KACs of SF-CSC. (*E*,*E*)-2,4-Heptadienal was the KAC in all the three CSC groups with no significant difference. (*E*)-2-Decenal increased significantly in SF-CSC, while octanal, (*E*)-2-nonenal, and benzaldehyde (cherry, nutty, almond) were newly generated after the CSC stir-frying.

Compared with CK-CSC, the aldehyde content in BL-CSC decreased by 46.51% but increased by 91.18% in SF-CSC (Figure 1b). Aldehydes were major flavor compounds in frying oil mainly generated by the oxidation of unsaturated fatty acids, and the content of the aldehydes increased linearly with the frying time [44]. This might be the main reason for the increase in aldehydes in SF-CSC.

#### 2.3.3. Ketones

Ketones are generated through amino acid catabolism and fat degradation, resulting in sweet, creamy, or buttery odors with low odor thresholds [33,45]. Compared with CK-CSC, the ketone content in BL-CSC decreased significantly by 58.30% and increased by 26.22% in SF-CSC (Figure 1b). Four ketones were detected in the CSC as shown in Table 1. Based on the OAV, (*E*)-β-ionone was the key aroma compound in CK-CSC and BL-CSC, and imparted the seaweed, violet, and fruity odor. As a terpene derivative, (*E*)-β-ionone occurs in red peppers and is generated by the degradation of carotenoids by LAB [46]. After cooking, the (*E*)-β-ionone was significantly decreased in BL-CSC and disappeared in SF-CSC. Similar results showed that the content of (*E*)-β-ionone in watermelon juice decreased significantly after heat treatment [14]. After stir-frying, 1-octen-3-one was newly generated, and contributed significantly to the flavor of SF-CSC and imparted the mushroom odor [47]. Adding peanut oil during the stir-frying process might be the main reason for the 1-octen-3-one increase in SF-CSC, as a previous study indicated that 1-octen-3-one was the main aroma compound in roasted peanuts, which was generated during peroxidation of unsaturated fatty acids [48].

#### 2.3.4. Acids

Acids are known to impart a sour odor to fermented vegetables. Compared with CK-CSC, the acid content in BL-CSC increased by 8.48%, and in SF-CSC increased by 157.31% (Figure 1b). Acetic acid, hexanoic acid, and octanoic acid were detected in all the CSC groups, while hexanoic acid was only detected in SF-CSC. Previous studies indicated that hexanoic acid was the major acid in frying oil at the end of the frying time [44]. And the formation of hexanoic acid was related to the β-oxidation of linoleic acid or the hydrolysis of ethyl hexanoate [49]. Acetic acid was the major acid in CSC with OAV > 1, which was mainly generated by LAB during CSC fermentation [20]. The concentration of acetic acid was significantly increased after boiling and stir-frying.

#### 2.3.5. Esters

Esters provide fruity, sweet, and floral flavor to fermented vegetables, generally originating from the esterification of short-chain acids with alcohols [33,46]. As shown in Table 1, four esters were detected in the CSC; however, esters may contribute limited influence on the CSC flavor due to the high odor threshold. Geranyl acetate and ethyl linolenate were newly generated, and geranyl acetate (sweet, fruity–floral) was the key aroma compound in BL-CSC with OAV > 1. Previous studies showed that geranyl acetate was the main flavor component of fresh baby ginger and contributed to the flavor with the highest relative content in baby ginger paocai, and it was also the main compound that contributed to the flavor characteristics of commercial pickles [34,50]. Geranyl acetate is insoluble in water, so boiling may be conducive to the dissolution and extraction of the compounds. No ester compounds were detected in SF-CSC, which suggested that ester compounds could be hydrolyzed to acids and alcohols at a high temperature, and this result was consistent with the increase in acid and alcohol content in CSC after cooking.

#### 2.3.6. Alcohols

Alcohols exhibit a sweet and floral flavor with a high threshold, and are essential for the production of esters and aldehydes [34]. Four alcohols were detected in the CSC samples, (*E*)-2-octen-1-ol in CK-CSC, linalool and geraniol in BL-CSC, and 1-octen-3-ol in SF-CSC, as shown in Table 1. (*E*)-2-Octen-1-ol had little effect on the flavor of CK-CSC due to a high threshold. (*E*)-2-Octen-1-ol was not detected after boiling, which was consistent with a previous study indicating that the content of (*E*)-2-octen-1-ol decreased sharply in the heating of watermelon juice [14]. Linalool and geraniol were newly generated after boiling, and linalool was identified as the KAC as OAV > 1. Linalool was the major compound in fermented chopped pepper and baby ginger paocai that contributed fruity, sweet, and floral aromas to BL-CSC [46,50]. 1-Octen-3-ol was newly generated after stir-frying with OAV = 51.28 giving a mushroom aroma to SF-CSC. It was suggested that the content of 1-octen-3-ol increased with the increase in temperature of peanut oil [51].

#### 2.3.7. Others

In total, five terpenes, β-sesquiphellandrene, zingiberene, β-bisabolene, farnesene, and curcumene, were detected in CSC samples, most of which were decreased significantly after cooking (Table 1). Most terpenes had little effect on the flavor of the three CSCs due to the high threshold. Previous studies showed that β-bisabolene, β-sesquiphellandrene, zingiberene, and curcumene were the main compounds in fresh baby ginger and ginger paocai [50]. By oxidative degradation, zingiberene and β-sesquiphellandrene were readily converted to curcumene [24].

Nitriles are important flavor compounds in fermented cruciferous plants and generated by glucosinolate degradation under acidic conditions [52]. Benzenepropanenitrile is a characteristic flavor component present in cruciferous plants with aldehydic and spicy odor [37]. It was the only nitrile compound detected in this study, the content of which was significantly decreased after boiling and remained constant after stir-frying.

### 2.4. Comparison of the KACs and Possible Pathways

**Figure 3:**
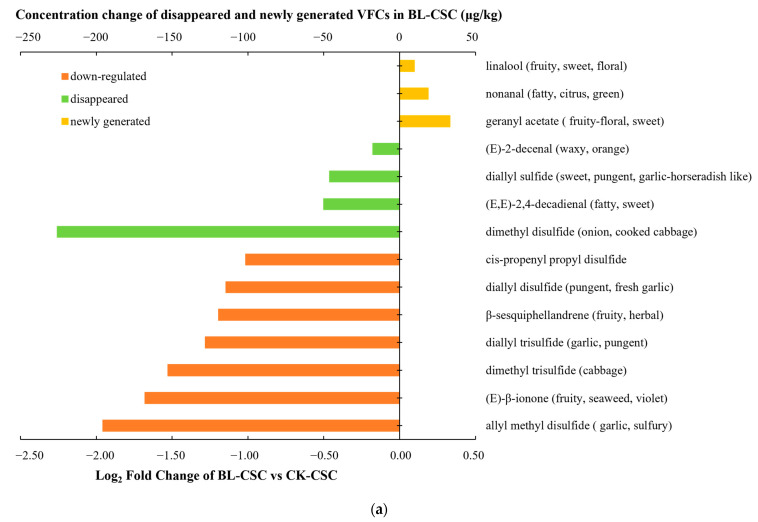

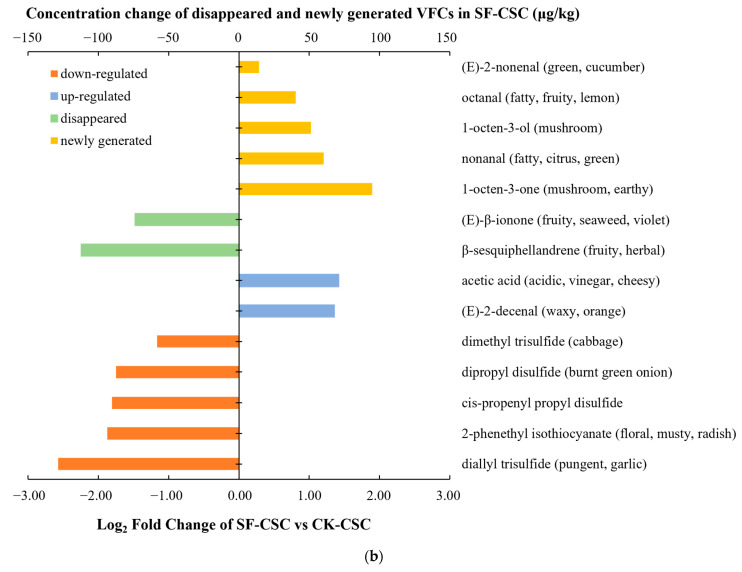

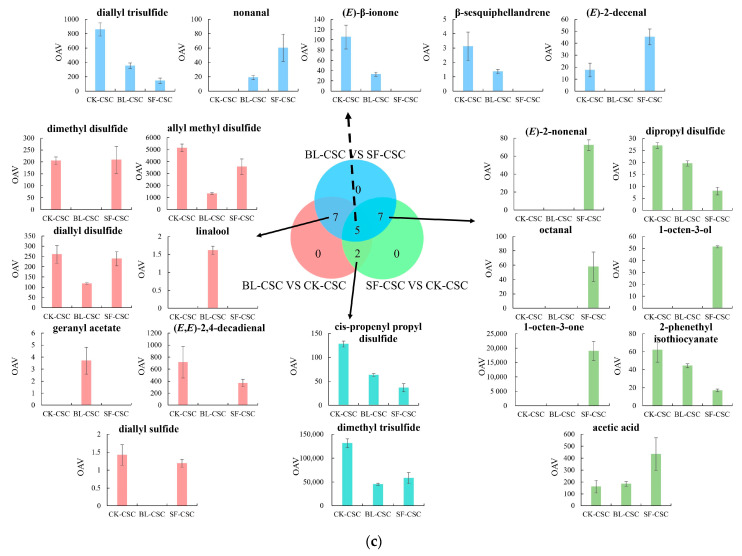
KACs (OAV > 1) in CSC samples under different cooking methods. (**a**) KACs with FC > 2 or <0.5, and newly generated or disappeared VFCs (BL-CSC vs. CK-CSC). (**b**) KACs with FC > 2 or <0.5, and disappeared or newly generated VFCs (SF-CSC vs. CK-CSC). (**c**) The potential markers responsible for the aroma differences and their OAV changes among the three CSC samples. The screening criteria were OAV > 1, and FC > 2 or <0.5, newly generated or disappeared. Abbreviations: KACs, key aroma compounds; OAV, odor activity value; CSC, Chinese spicy cabbage; FC, fold change; VFCs, volatile flavor compounds; CK-CSC, the CSC without thermal processing group; BL-CSC, boiled CSC; SF-CSC, stir-fried CSC.

To further distinguish the main flavor compounds and their conveyed odors between different groups, the KAC differences among the three CSC samples were further screened based on the analysis of OAV (OAV > 1) and fold change (FC > 2 or <0.5). The histograms show the VFCs with greater than two-fold change, and the newly generated or disappeared (Figure 3a,b), indicating a significant change in flavor compounds after cooking.

Comparing BL-CSC with CK-CSC (Figure 3a), three KACs (linalool, geranyl acetate, and nonanal) were newly generated after boiling, contributing sweet and fatty odors to BL-CSC. Meanwhile, four KACs including (*E*)-2-decenal, diallyl sulfide, (*E*,*E*)-2,4-decadienal, and dimethyl disulfide disappeared after boiling, resulting in the lack of waxy, sweet, and onion odor compared with CK-CSC. And seven KACs including *cis*-propenyl propyl disulfide, diallyl disulfide, β-sesquiphellandrene, diallyl trisulfide, dimethyl trisulfide, (*E*)-β-ionone, and allyl methyl disulfide were significantly reduced (FC < 0.5) in BL-CSC, which reduced the pungent, herbal, garlic, cabbage, and seaweed odor to a certain extent. Comparing SF-CSC with CK-CSC (Figure 3b), the newly generated (*E*)-2-nonanal, octanal, 1-octen-3-ol, nonanal, and 1-octen-3-one after stir-frying gave SF-CSC green, fatty, and mushroom odors. Acetic acid and (*E*)-2-decenal with FC > 2 increased the SF-CSC acidic and orange odor contrasted with CK-CSC. Meanwhile, (*E*)-β-ionone and β-sesquiphellandrene disappeared resulting in reduced fruity odor of SF-CSC. And five KACs including dimethyl trisulfide, dipropyl disulfide, cis-propenyl propyl disulfide, 2-phenethyl isothiocyanate, and diallyl trisulfide that decreased by greater than two-fold were all sulfides, which would reduce the cabbage, burnt green onion, radish, pungent, and garlic odor in SF-CSC. The KACs increased and newly generated in SF-CSC were more than those in BL-CSC, with more abundant odor changes, which was due to higher cooking temperature, and the peanut oil added in the stir-frying process that was related to the increase of aldehydes, acids, and alcohols [44,51,53].

The potential markers causing aroma differences among the three CSC samples were screened by analyzing VFCs with OAV > 1, and FC > 2 or <0.5, newly generated or disappeared as shown in Figure 3c. Five VFCs were selected as the potential KACs to distinguish the different CSC samples, including diallyl trisulfide, nonanal, (*E*)-β-ionone, β-sesquiphellandrene, and (*E*)-2-decenal.

**Figure 4:**
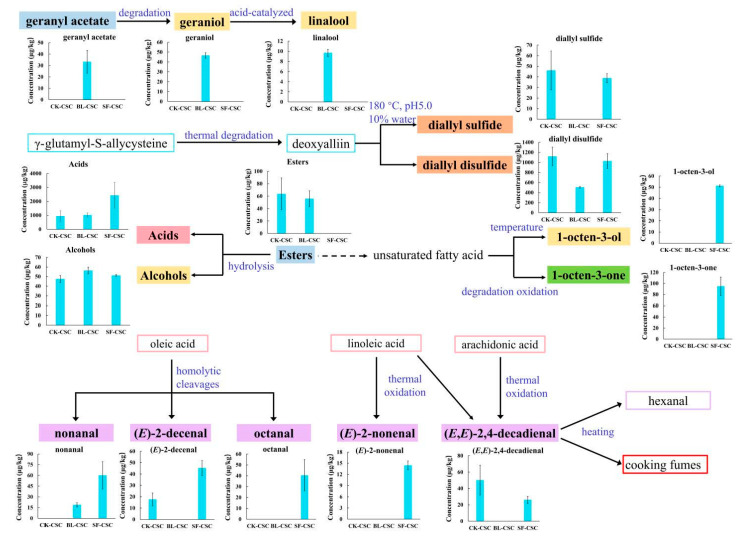
Possible formation pathways of key aroma compounds (KACs) after heat treatment.

The possible formation pathways of KACs after cooking are shown in Figure 4. It was suggested that boiling may facilitate the dissolution and extraction of geranyl acetate, leading to its increase in BL-CSC. Geraniol was produced by thermal degradation of geranyl acetate under acidic conditions and the main product from geraniol during acid-catalyzed transformation was linalool [54]. Sulfides mainly originated from degradation of sulfur-containing amino acids in vegetables, also allicin and deoxyalliin in garlic [24,55]. The reduction in sulfides might be due to the thermal treatment. However, the contents of diallyl sulfide and diallyl disulfide in SF-CSC were significantly higher than those in BL-CSC, which indicated that diallyl sulfide and diallyl disulfide could be generated from the degradation of deoxyalliin in 10% water, pH 5.0, at 180 °C [56]. Esters could be hydrolyzed to acids and alcohols at high temperatures, which resulted in the decrease in esters, and the increase in acids and alcohols in CSC after cooking. 1-Octen-3-ol in heated peanut oil and 1-octen-3-one in roasted peanuts were generated from the peroxidation of unsaturated fatty acids [48,51]. The nonanal, (*E*)-2-decenal, octanal, and (*E*)-2-nonenal increases in SF-CSC were generated from the oxidation of oleic acid and linoleic acid in peanut oil [57]. However, (*E*,*E*)-2,4-decadienal was generated from linoleic acid and arachidonic acid in peanut oil after stir-frying, and transferred to cooking fumes, resulting in a decrease in CSC after cooking [58].

### 2.5. Odor Profiles

**Figure 5:**
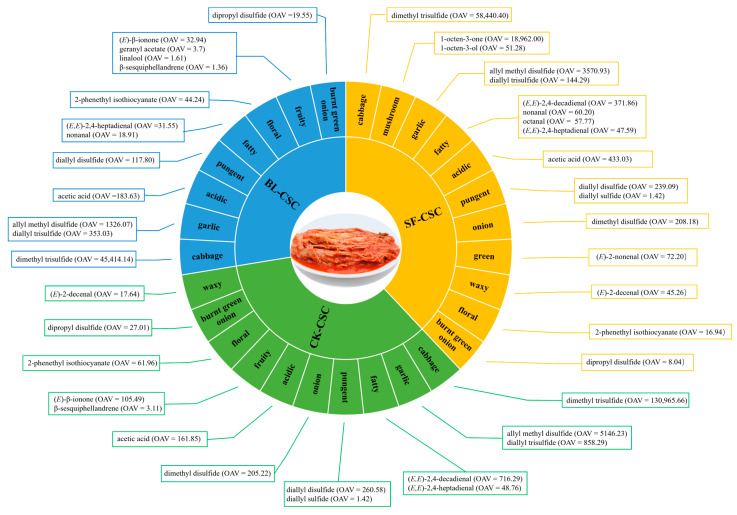
Odor wheel of different CSC samples. Abbreviations: CSC, Chinese spicy cabbage; CK-CSC, the CSC without thermal processing group; BL-CSC, boiled CSC; SF-CSC, stir-fried CSC; OAV, odor activity value.

In order to distinguish the odor profiles among different CSC groups, a total of 20 KACs with OAV > 1 and definite odor description were selected to form an odor wheel as shown in Figure 5. Among all KACs, dimethyl trisulfide with the highest OAV in the three CSCs contributed cabbage odor. Sulfides of allyl methyl disulfide, diallyl trisulfide, diallyl disulfide, and dimethyl disulfide with OAV > 100 contributed the main odors of garlic, pungent, and onion in the three CSCs. Acetic acid presented acidic and vinegar odors in all the CSC groups with OAV > 150, and the odor intensity enhanced after cooking. (*E*)-β-Ionone was the main KAC contributing a fruity and seaweed odor in CK-CSC with OAV = 105.49, and the odor intensity decreased in BL-CSC with OAV = 32.94, disappearing in SF-CSC. Aldehydes including (*E,E*)-2,4-decadienal (OAV > 300), nonanal (OAV > 60), octanal (OAV > 50), and (*E,E*)-2,4-heptadienal (OAV > 40) mainly presented fatty odors in SF-CSC. 1-Octen-3-one (OAV = 18,962) and 1-octen-3-ol (OAV = 51.28) presented a mushroom odor in SF-CSC, which was the unique flavor of CSC after stir-frying.

The overall flavors of CSCs were weakened after cooking, as the total OAV values decreased significantly in BL-CSC (total OAV = 47,548.57) and SF-CSC (total OAV = 82,730.25), compared with CK-CSC (total OAV = 138,579.51). As shown in Figure 5, a total of 14 KACs selected in CK-CSC contributed cabbage, garlic, fatty, pungent, onion, acidic, fruity, floral, burnt green onion, and waxy odors in order of OAV value and odor intensity. In BL-CSC, in total 13 KACs mainly contributed cabbage, garlic, acidic, pungent, fatty, floral, fruity, and burnt green onion odors. Compared with CK-CSC, the onion and waxy odors disappeared, acidic and pungent odors were more prominent, and fatty odor was decreased. In SF-CSC, in total 17 KACs mainly contributed cabbage, mushroom, garlic, fatty, acidic, pungent, onion, garlic, green, waxy, floral, and burnt green onion odors. Compared with CK-CSC, the mushroom and green odors were newly generated in SF-CSC, and mushroom odor was prominent. Meanwhile, fruity odor disappeared, and acidic and waxy odors were increased in SF-CSC.

### 2.6. Sensory Evaluation

Glossiness is an indicator of CSC appearance, which reflects the organoleptic property that affects consumers’ appetite. The CSCs after cooking were characterized by a lower perception of glossiness than CK-CSC. The browning of BL-CSC and SF-CSC were caused by thermal processes, which might be due to the sugar–sugar reactions at high temperatures, or the Maillard reactions between reducing sugars and ammonia compounds, such as proteins, amino acids, and amides [19].

**Figure 6:**
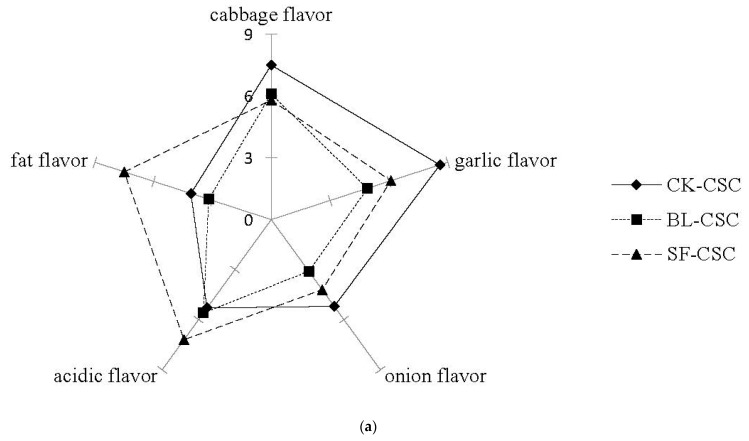

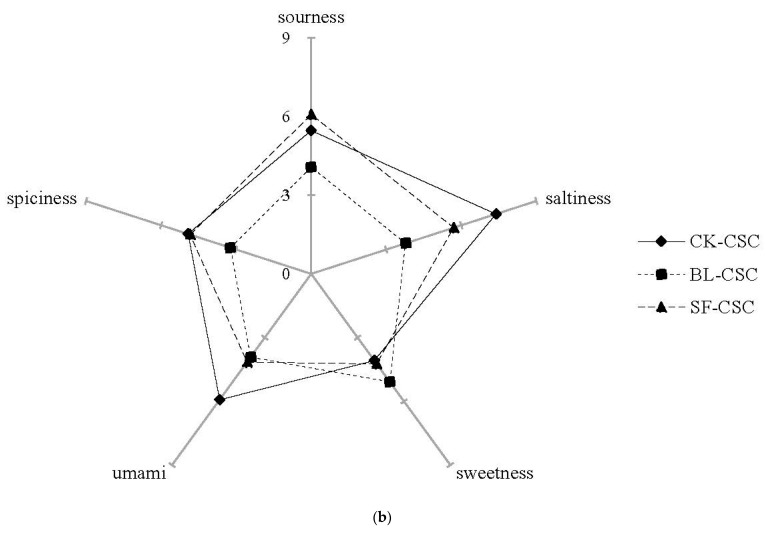
Sensory characteristics under different cooking methods. (**a**) Aroma properties of CSCs by QDA. (**b**) Taste qualities of CSCs by QDA. Abbreviations: QDA, quantitative descriptive analysis; CSC, Chinese spicy cabbage; CK-CSC, the CSC without thermal processing group; BL-CSC, boiled CSC; SF-CSC, stir-fried CSC.

In addition to appearance, aroma was an important sensory attribute in determining the consumer acceptance of CSC. Figure 6a shows the aroma evaluation of CSC samples after cooking differed in five flavor qualities. The CK-CSC was characterized by a higher perception of cabbage, garlic, and onion flavor, due to the higher content of the sulfides. The flavors of cabbage, garlic, and onion in CSCs after cooking were weakened, which was due to the significant decrease in sulfide contents in BL-CSC and SF-CSC. The flavor sense of BL-CSC was relatively moderate or weak, which might be due to the loss of flavor compounds during heat treatment. The SF-CSC was characterized by a higher perception of fat and acidic flavor, mainly because of the peanut oil added in the stir-frying processing, and the aldehydes and acids generated at high temperature. Although the total OAVs of aldehydes with fatty flavor in CK-CSC was higher than that of SF-CSC, the fat flavor in SF-CSC was stronger by sensory evaluation. That might be because the types of aldehydes detected in SF-CSC were more abundant, leading to the mutual enhancement effect of fat flavor among different aldehydes. Understanding the effects of different cooking processes on the changes in the VFCs in CSC would help to explain their influence on sensory profiles.

Five taste qualities (sourness, saltiness, sweetness, umami, and spiciness) of CSCs were evaluated as shown in Figure 6b. The CK-CSC was characterized by a higher perception of saltiness and umami, and moderate intensities of sourness. The SF-CSC was characterized by a higher perception of sourness and moderate saltiness. The higher perception of sourness in SF-CSC was consistent with acids increasing in SF-CSC detected by TTA analysis and GC-MS. Besides a higher perception of sweetness, the BL-CSC was characterized by a lower perception of sourness, saltiness, and spiciness. The stronger sweetness of BL-CSC might be due to the hydrolysis and release of saccharides during heat processing. The decrease in taste property intensity of CSC after cooking might be due to the loss of some nutrients and flavor compounds during heat processing.

## 3. Materials and Methods

### 3.1. Preparation of Chinese Spicy Cabbage

Chinese cabbage (*Brassica rapa* ssp. *pekinensis*) and other materials were purchased from a local supermarket in Yantai, Shandong Province, China. CSC was produced according to Xing, et al. [2]. The fresh Chinese cabbage was marinated with 10% salt water (salt water: cabbage = 1:1) for 12 h. The pickled cabbage was rinsed with running water for 10 s, drained of water for about 1 h, and then cut into 3 × 3 cm pieces and mixed with seasonings as follows: salted cabbage (72.1%), white radish (12.0%), onion (3.6%), garlic (3.6%), glutinous rice paste (3.6%), red pepper (1.7%), shrimp sauce (1.2%), fish sauce (1.2%), ginger (0.5%), and sucrose (0.5%). The seasoned samples were placed into a 10 L ceramic jar and then fermented at 10 °C for 7 d.

### 3.2. Cooking Process

The cooking methods were carried out in triplicate according to Pérez, et al. [59] with modifications. For the boiled CSC group (BL-CSC), 100 g CSC was immersed in 100 g boiling water in a stockpot for 10 min. After boiling, sterilized water was added to 200 g for analysis. For the stir-fried CSC group (SF-CSC), 16 g peanut oil was preheated for 1 min in a frying pan until the oil temperature reached 180 °C. Then 100 g CSC was added to the frying pan and stir-fried for 3 min in the preheated oil until the sample became crisp-tender. The CSC without cooking was set as control (CK-CSC).

Forty grams of CK-CSC or SF-CSC samples were taken, supplemented with the same amount of sterile water, and then homogenized with a stirrer. Eighty grams of BL-CSC was homogenized with a stirrer for further analysis. Physicochemical properties and flavor compounds were analyzed in triplicate.

### 3.3. TTA Analysis

The TTA of CSC samples was measured following the AOAC [60] official method. The homogenized sample (30 g) was filtered through sterile gauze. Fifteen grams filtrate and 30 mL sterile water were mixed and titrated using 0.1 N NaOH to a final pH of 8.3. TTA was expressed in percentage of lactic acid using the following formula:

$$\mathrm{TTA}\;\left(\%\right)=\frac{\mathrm{Volume}\;\mathrm{of}\;0.1\;N\;\mathrm{NaOH}\;\left(\mathrm{mL}\right)\times 0.1\;N\;\mathrm{NaOH}\times 0.090\times 100}{\mathrm{Sample}\;\left(g\right)}$$

### 3.4. Total Sugar

Total sugar was analyzed according to Masuko, et al. [61] with some modifications. Homogenized sample solution (0.2 mL) was mixed with 1 mL anthrone–sulfuric acid reagent, and boiled for 10 min. The final reaction solutions (200 μL) were added into a 96-well plate, and the absorbance was measured at 620 nm using a microplate spectrophotometer (Spectramax 190, Molecular Devices Corporation, San Jose, CA, USA). The results were calculated by the standard curve obtained from 0.01 to 0.1 mg/mL of glucose solutions.

### 3.5. Sensory Evaluation

Sensory profiles were evaluated by quantitative descriptive analysis (QDA) according to Choi, et al. [20] with modifications. Sensory evaluation of appearance (glossiness), aroma (cabbage flavor, garlic flavor, onion flavor, acidic flavor, and fat flavor), and taste (sourness, saltiness, sweetness, umami, and spiciness) were evaluated by 13 trained assessors using a 9-point scale from 1 (very low) to 9 (very strong). Thirteen assessors made up of 7 females and 6 males aged 18–42 were selected to form a sensory panel. Twenty grams of each sample was provided in white polyethylene cups and numbered with three digits randomly. Water and crackers were used to refresh their palates between samples.

### 3.6. GC-MS Analysis

For volatile compound analysis, 2 g of CSC sample was put into a headspace vial with 15 μL (0.01 mg/mL) 3-nonanone (internal standard), and stirred (300 rpm) at 70 °C for 10 min. Volatiles were adsorbed using divinylbenzene/carboxen/polydimethylsiloxane (DVB/CAR/PDMS) fiber with a thickness of 50/30 µm for 30 min. After extraction, the fiber was introduced into the chromatograph for desorption at 250 °C for 5 min in split mode. GC-MS system (GC-MS-QP2020 NX, Shimadzu Corporation, Kyoto, Japan) with SH stabilwax column (60 m × 0.25 mm × 0.25 μm) was used for analysis. Helium (1 mL/min) was used as the carrier gas. The GC was programmed as follows: 40 °C holding for 3 min, increasing the temperature to 115 °C at 5 °C/min, increasing to 150 °C at 2 °C/min, increasing to 230 °C at 7 °C/min, and holding for 10 min. The mass spectrometry was performed at 70 eV in electron impact ionization mode with a scan range of 50~550 *m*/*z*. The linear retention index (LRI) of volatile compounds was calculated based on the series of *n*-alkanes (C_10_–C_40_). The mass spectra of the volatile compounds were compared to those in the NIST 17 mass spectral library or in previously published articles. By comparing the values of the LRI with those of published values, identities were confirmed whenever possible. The relative concentration of each volatile compound was quantified as equivalent to the internal standard by the peak area.

### 3.7. Statistical Analysis

All experimental data are displayed as mean ± SD (*n* = 3). The differences among groups were determined by one-way analysis of variance (ANOVA) with Duncan’s multiple range test (*p* < 0.05) using SPSS statistics version 24 (SPSS Inc., Chicago, IL, USA). Principal component analysis (PCA) and the heatmap were obtained using TBtools software (version 0.66837, Nanjing, China). The OAVs of flavor compounds were calculated according to Yang, et al. [32].

## 4. Conclusions

Two cooking methods of boiling and stir-frying had a great influence on the physicochemical properties, flavor components, and sensory qualities of CSC. Compared with CK-CSC, the TTA of CSCs after cooking significantly increased; however, the total sugar contents significantly decreased after stir-frying. A total of 47 VFCs were detected in CSC by GC-MS, and 21 VFCs with OAV > 1 and definite odor description were selected as the KACs including 8 sulfides, 6 aldehydes, 2 ketones, 1 acid, 1 ester, 2 alcohols, and 1 terpene. Compared with CK-CSC, seven KACs decreased significantly, four KACs disappeared, and three KACs were newly generated in BL-CSC, resulting in the disappearance of onion and waxy odors, more prominent acidic and pungent odors, and reduced fatty odor. However, in the SF-CSC group, two KACs increased and five KACs decreased significantly, two KACs disappeared, and five KACs were newly generated, which mainly resulted in the decrease in cabbage, garlic, pungent, fruity, floral, and burnt green onion odors, and increase in mushroom, acidic, green, and waxy odors. Although the total OAV of KACs exhibiting fatty odor was reduced in SF-CSC, the fat flavor in SF-CSC was stronger by sensory evaluation than that of CK-CSC, which might be due to the abundance of aldehydes detected in SF-CSC, leading to mutual enhancement of fat flavors between different aldehydes. The contents of diallyl trisulfide, nonanal, (*E*)-β-ionone, β-sesquiphellandrene, and (*E*)-2-decenal changed greatly among the three CSC groups, so these five KACs were identified as the characteristic potential markers of aroma differences.

Through different cooking methods, some beneficial flavor compounds were newly generated or increased, contributing abundant flavors to CSC. However, cooking caused browning of CSC and a reduction in characteristic sulfides. Therefore, on the basis of traditional cooking, it is necessary to combine cold processing methods such as high-pressure and microwave to inactivate microorganisms and enzymes to retain more flavors of CSC. In addition, the parameters in the cooking process could be further studied to develop more diverse and flavorful CSC products. This study also provides a reference for consumers in choosing different CSC products, and contributes to the industrial production of higher quality and more convenient CSC products.

## Figures and Tables

**Table 1:** The volatile compounds of CSC identified by GC-MS.

Number	Compounds	LRI ^a^	LRI ^b^	Odor Threshold (μg/kg)	Concentration (μg/kg, CK-CSC)	OAV (CK-CSC)	Concentration (μg/kg, BL-CSC)	OAV (BL-CSC)	Concentration (μg/kg, SF-CSC)	OAV (SF-CSC)	Odor Description
**Sulfides (15)**											
1	Diallyl sulfide	1146	1148 ± 3	32.5 [21]	46.11 ± 9.36 ^a^	1.42	n.d.		38.80 ± 3.45 ^a^	1.19	Pungent, sweet, Garlic–horseradish-like [22]
2	Dimethyl disulfide	1078	1077 ± 8	1.1 [23]	225.74 ± 17.44 ^a^	205.22	n.d.		229 ± 62.44 ^a^	208.18	Onion, cooked cabbage [23]
3	Methyl propyl disulfide	1233	1239 ± 13		66.71 ± 5.48 ^a^		n.d.		38.18 ± 3.93 ^b^		Garlic, sour [24]
4	Allyl methyl disulfide	1285	1281 ± 10	0.3 [25]	1543.87 ± 96.53 ^a^	5146.23	397.82 ± 20.65 ^c^	1326.07	1071.28 ± 198.03 ^b^	3570.93	Garlic, sulfury [22]
5	Methyl *cis*-propenyl disulfide	1299	1303 ± 5		207.70 ± 25.44 ^a^		50.83 ± 5.95 ^c^		92.14 ± 12.22 ^b^		Garlic [24]
6	Dipropyl disulfide	1383	1378 ± 11	3.2 [26]	86.43 ± 3.98 ^a^	27.01	62.57 ± 3.59 ^b^	19.55	25.74 ± 5.13 ^c^	8.04	Burnt green onion [27]
7	*cis*-Propenyl propyl disulfide	1436	1421 ± 15	2 [26]	255.34 ± 12.34 ^a^	127.67	126.40 ± 6.18 ^b^	63.20	73.15 ± 17.12 ^c^	36.58	
8	Diallyl disulfide	1488	1475 ± 14	4.3 [25]	1120.50 ± 184.75 ^a^	260.58	506.52 ± 16.65 ^b^	117.8	1028.07 ± 147.04 ^a^	239.09	Pungent, fresh garlic [25]
9	3-Vinyl-1,2-dithi-4-ene	1750	1750 ± N/A		66.09 ± 12.70 ^a^		n.d.		n.d.		Garlic, pungent [28]
10	Dimethyl trisulfide	1388	1377 ± 11	0.0099 [25]	1296.56 ± 92.64 ^a^	130,965.66	449.60 ± 22.99 ^b^	45,414.14	578.56 ± 115.07 ^b^	58,440.40	Cabbage [25]
11	Allyl methyl trisulfide	1594	1593 ± 1		1131.95 ± 186.52 ^a^		423.38 ± 22.42 ^b^		641.48 ± 99.38 ^b^		Sulfury, eggy [25]
12	Allyl propyl trisulfide	1737	1760 ± 37		73.72 ± 3.43 ^a^		52.15 ± 6.09 ^b^		n.d.		
13	Diallyl trisulfide	1794	1805 ± 16	0.34 [25]	291.82 ± 30.88 ^a^	858.29	120.03 ± 13.32 ^b^	353.03	49.06 ± 12.95 ^c^	144.29	Garlic, pungent [25]
14	2-Butyl isothiocyanate	1287	1287 ± N/A		39.05 ± 1.61 ^a^		14.25 ± 1.00 ^b^		35.81 ± 6.63 ^a^		
15	2-Phenethyl isothiocyanate	2234	2234 ± 0	10 [29]	619.58 ± 137.41 ^a^	61.96	442.43 ± 22.35 ^a^	44.24	169.36 ± 15.40 ^b^	16.94	Floral, musty, radish [24]
**Aldehydes (10)**											
16	(*E*)-2-Heptenal	1328	1323 ± 11	13 [21]	n.d.		7.77 ± 1.13 ^a^	<1	n.d.		Fatty, almond, green [14]
17	(*E,E*)-2,4-Heptadienal	1503	1495 ± 11	1 [30]	48.76 ± 17.36 ^a^	48.76	31.55 ± 3.53 ^a^	31.55	47.59 ± 10.18 ^a^	47.59	Fatty, nutty, green [30]
18	Octanal	1289	1289 ± 9	0.7 [21]	n.d.		n.d.		40.44 ± 14.32 ^a^	57.77	Fatty, fruity, lemon [14]
19	Nonanal	1395	1391 ± 8	1 [21]	n.d.		18.91 ± 3.02 ^b^	18.91	60.20 ± 19.00 ^a^	60.20	Fatty, citrus, green [14]
20	(*E*)-2-Nonenal	1542	1534 ± 10	0.2 [14]	n.d.		n.d.		14.44 ± 1.19 ^a^	72.20	Green, cucumber [14]
21	(*E*)-2-Decenal	1649	1644 ± 11	1 [30]	17.64 ± 5.67 ^b^	17.64	n.d.		45.26 ± 6.65 ^a^	45.26	Waxy, orange [30]
22	(*E*,*E*)-2,4-Decadienal	1818	1811 ± 16	0.07 [21]	50.14 ± 18.26 ^a^	716.29	n.d.		26.03 ± 4.30 ^b^	371.86	Fatty, sweet [24]
23	1-Pentadecanal	2041	2041 ± 0	430 [31]	13.66 ± 3.46 ^a^	<1	5.35 ± 1.05 ^b^	<1	n.d.		Metal, plastic [31]
24	Benzaldehyde	1534	1520 ± 14	350 [21]	n.d.		n.d.		55.69 ± 8.76 ^a^	<1	Cherry, nutty, almond [14]
25	2,4-Dimethylbenzaldehyde	1725	1726 ± 16	1000 [14]	28.38 ± 5.63 ^a^	<1	21.24 ± 8.48 ^a^	<1	13.53 ± 6.62 ^a^	<1	Bitter almond [14]
**Ketones (4)**											
26	1-Octen-3-one	1302	1300 ± 8	0.005 [21]	n.d.		n.d.		94.81 ± 16.81 ^a^	18,962.00	Mushroom, earthy [24]
27	Geranyl acetone	1861	1859 ± 9	60 [21]	34.34 ± 9.04 ^a^	<1	22.05 ± 7.54 ^a^	<1	n.d.		Floral, green [32]
28	(*E*)-β-Ionone	1947	1940 ± 17	0.7 [14]	73.84 ± 16.22 ^a^	105.49	23.06 ± 2.73 ^b^	32.94	n.d.		Fruity, seaweed, violet [14]
29	4-Hydroxy-3-methylacetophenone	2213	2210 ± 31		n.d.		n.d.		41.74 ± 8.38 ^a^		
**Acids (3)**											
30	Acetic acid	1456	1449 ± 13	5.5 [23]	890.15 ± 290.45 ^b^	161.85	1010.19 ± 118.51 ^b^	183.67	2381.65 ± 752.05 ^a^	433.03	Acidic, vinegar, cheesy [23]
31	Hexanoic acid	1856	1846 ± 12	3000 [21]	n.d.		n.d.		29.11 ± 4.89 ^a^	<1	Sweaty, cheesy, sharp, goaty, bad breath [33]
32	Octanoic acid	2065	2060 ± 15	3000 [21]	56.45 ± 14.16 ^a^	<1	16.71 ± 2.48 ^b^	<1	24.92 ± 4.15 ^b^	<1	Sweat, cheese [33]
**Esters (4)**											
33	Geranyl acetate	1755	1752 ± 11	9 [21]	n.d.		33.27 ± 10.00 ^a^	3.70	n.d.		Fruity–floral, sweet [34]
34	Methyl palmitate	2218	2208 ± 10	4 × 10^6^ [35]	34.52 ± 10.94 ^a^	<1	n.d.		n.d.		Oily, waxy, fatty [35]
35	Ethyl palmitate	2257	2251 ± 9	2000 [35]	29.28 ± 10.04 ^a^	<1	16.85 ± 2.07 ^a^	<1	n.d.		Fatty, fruity, waxy [35]
36	Ethyl linolenate	2604	2591 ± 13	450 [35]	n.d.		5.98 ± 0.61 ^a^	<1	n.d.		Fatty, waxy [35]
**Alcohols (4)**											
37	1-Octen-3-ol	1148	1450 ± 7	1 [21]	n.d.		n.d.		51.28 ± 0.86 ^a^	51.28	Mushroom [14]
38	(*E*)-2-Octen-1-ol	1615	1614 ± 7	100 [14]	47.48 ± 3.53 ^a^	<1	n.d.		n.d.		Green, soap, plastic [14]
39	Linalool	1544	1547 ± 7	6 [21]	n.d.		9.68 ± 0.71 ^a^	1.61	n.d.		Fruity, sweet, floral [33]
40	Geraniol	1852	1847 ± 10	40–75 [21]	n.d.		46.68 ± 2.78 ^a^	<1	n.d.		Sweet rose, pleasant, floral [34]
**Terpenes (5)**											
41	β-Sesquiphellandrene	1773	1772 ± 8	36 [36]	112.02 ± 35.92 ^a^	3.11	48.98 ± 5.53 ^b^	1.36	n.d.		Fruity, herbal [36]
42	Zingiberene	1723	1724 ± 9		170.58 ± 48.77 ^a^		63.60 ± 12.44 ^b^		17.68 ± 4.48 ^b^		Spice, fresh, sharp [36]
43	β-Bisabolene	1729	1727 ± 11		80.63 ± 20.22 ^a^		33.43 ± 3.09 ^b^		n.d.		Balsamic, woody [36]
44	Farnesene	1744	1746 ± 9		n.d.		12.34 ± 0.23 ^a^		n.d.		Citrus, herbal, lavender, bergamot [36]
45	Curcumene	1778	1777 ± 9		284.92 ± 111.48 ^a^		111.25 ± 15.00 ^b^		13.00 ± 2.42 ^b^		Herbal [36]
**Nitriles (1)**											
46	Benzenepropanenitrile	2047	2041 ± 7		1317.01 ± 207.72 ^a^		763.87 ± 33.90 ^b^		1703.69 ± 321.51 ^a^		Aldehydic, spicy [37]
**Phenols (1)**											
47	2,4-Di-tert-butylphenol	2316	2318 ± 10		n.d.		30.62 ± 10.89 ^a^		n.d.		

(1) The concentrations are the means of three repetitions ± standard deviation. (2) Different letters for the same row indicate significant differences according to Duncan’s multiple range test (*p* < 0.05). (3) LRI ^a^: linear retention index calculated by a homologous series of *n*-alkanes (C_10_–C_40_). (4) LRI ^b^: linear retention index of reference compounds in NIST library and literature. (5) n.d.: Not detected. (6) OAV were calculated by dividing the average concentration by the odor threshold. (7) Aroma-active compounds identified by OAV and GC-MS in CSC sample. (8) Abbreviations: CSC, Chinese spicy cabbage; CK-CSC, the CSC without thermal processing group; BL-CSC, boiled CSC; SF-CSC, stir-fried CSC; OAV, odor activity value.

## Data Availability

All the data are available within the manuscript.
